# Asymptomatic *Leishmania infantum* infection in dogs and dog owners in an endemic area in southeast France[Fn FN1]

**DOI:** 10.1051/parasite/2024019

**Published:** 2024-03-26

**Authors:** Mallorie Hide, Gregory Michel, Kevin Legueult, Raphaelle Pin, Susana Leonard, Loïc Simon, Anne-Laure Bañuls, Pascal Delaunay, Pierre Marty, Christelle Pomares

**Affiliations:** 1 MIVEGEC, Université de Montpellier, IRD, CNRS 911 av Agropolis 34090 Montpellier France; 2 Centre Méditerranéen de Médecine Moléculaire (C3M), U1065, Université Côte d’Azur, Inserm 151 route Saint Antoine de Ginestière BP 2 3194 06204 Nice France; 3 Département de Santé Publique, UR2CA, Université Côte d’Azur, Centre Hospitalier Universitaire de Nice 151, route de Saint Antoine de Ginestière CS 23079 06202 Nice France; 4 Laboratoire Vétérinaire Départemental 105 route des Chappes BP 107 06902 Sophia-Antipolis France; 5 Service de Parasitologie Mycologie, CHU Nice 151, route de Saint Antoine de Ginestière CS 23079 06202 Nice France

**Keywords:** Asymptomatic *Leishmania* infection, Humans, Dogs, kDNA PCR, ELISA, Western Blot

## Abstract

The prevalence of asymptomatic leishmaniasis in dogs and their owners in the main endemic areas of France has not been studied to date. The objective of this study was to quantify asymptomatic *Leishmania infantum* infection in southeast France in healthy people and their dogs using molecular and serological screening techniques. We examined the presence of parasitic DNA using specific PCR targeting kinetoplast DNA (kDNA) and specific antibodies by serology (ELISA for dogs and Western blot for humans) among immunocompetent residents and their dogs in the Alpes-Maritimes. Results from 343 humans and 607 dogs were included. 46.9% (*n* = 161/343) of humans and 18.3% (*n* = 111/607) of dogs were PCR positive; 40.2% of humans (*n* = 138/343) and 9.9% of dogs (*n* = 60/607) were serology positive. Altogether, 66.2% of humans (*n* = 227) and 25.7% of dogs (*n* = 156) had positive serologies and/or positive PCR test results. Short-haired dogs were more frequently infected (71.8%, *n* = 112) than long-haired dogs (12.2%, *n* = 19) (*p* = 0.043). Dogs seemed to be more susceptible to asymptomatic infection according to their breed types (higher infection rates in scenthounds, gun dogs and herding dogs) (*p* = 0.04). The highest proportion of dogs and human asymptomatic infections was found in the Vence Region, corresponding to 28.2% (*n* = 20/71) of dogs and 70.5% (*n* = 31/44) of humans (4.5/100,000 people). In conclusion, the percentage of infections in asymptomatic humans is higher than in asymptomatic dogs in the studied endemic area. It is questionable whether asymptomatic infection in humans constitutes a risk factor for dogs.

## Abstract (fr)

La prévalence de la leishmaniose asymptomatique chez les chiens et leurs propriétaires dans les principales zones d’endémie françaises n’a pas été étudiée à ce jour. L’objectif de cette étude était de quantifier l’infection asymptomatique à *Leishmania infantum* dans le sud-est de la France chez des personnes saines et leurs chiens à l’aide de techniques de dépistage moléculaire et sérologique. Nous avons examiné chez des résidents immunocompétents et leurs chiens dans les Alpes-Maritimes la présence d’ADN parasitaire par PCR spécifique ciblant l’ADN du kinétoplaste (ADNk) et d’anticorps spécifiques par sérologie (ELISA pour le chien et Western Blot pour l’homme). Les résultats de 343 humains et 607 chiens ont été inclus; 46,9 % (*n* = 161/343) des humains et 18,3 % (*n* = 111/607) des chiens étaient positifs à la PCR et 40,2 % des humains (*n* = 138/343) et 9,9 % des chiens (*n* = 60/607) avaient une sérologie positive. Au total, 66,2 % des humains (*n* = 227) et 25,7 % des chiens (*n* = 156) avaient des sérologies positives et/ou des résultats de tests PCR positifs. Les chiens à poils courts étaient plus fréquemment infectés (71,8 %, *n* = 112) que les chiens à poils longs (12,2 %, *n* = 19) (*p* = 0,043). Les chiens semblaient plus sensibles à l’infection asymptomatique selon leurs races (taux supérieurs chez les chiens de chasse et chiens de berger) (*p* = 0,04). La plus forte proportion d’infections asymptomatiques chez les chiens et les humains a été observée dans la Région de Vence, correspondant à 28,2 % (*n* = 20/71) des chiens et 70,5 % (*n* = 31/44) des humains (4,5/100 000). personnes). En conclusion, le pourcentage d’infections chez les humains asymptomatiques est plus élevé que chez les chiens asymptomatiques dans la zone d’endémie étudiée. On peut se demander si une infection asymptomatique chez l’homme constitue un facteur de risque pour les chiens.

## Introduction

Leishmaniases are caused by *Leishmania* spp., hemoflagellate protozoa belonging to the order Trypanosomatidae. These parasites are transmitted by the bite of infected phlebotomine sand flies. By the end of the last century, epidemiologic studies in several countries (Iran, Brazil, Spain, Italy and France) had shown that the prevalence of asymptomatic human carriers of *Leishmania* spp. is actually significant [2, 4, 7, 13, 25, 28, 33]. These patients do not have clinical signs in favor of visceral leishmaniasis (VL), but parasitic DNA is detected in peripheral blood [2, 7, 13, 25, 38]. On the Mediterranean coast, *Leishmania infantum* is the etiological agent of leishmaniasis. In humans, the majority of infections with *L. infantum* remain asymptomatic [6], but a minority of cases evolve to classic VL; however cutaneous and mucocutaneous infections are also possible [10]. *Leishmania infantum* is also responsible for canine leishmaniasis (CanL) for which dogs are the main reservoir. Dogs can remain asymptomatic for a certain period, but may develop a symptomatic form sooner or later during their lifetime [14, 15, 21]. Both asymptomatic and symptomatic dogs are *L. infantum* reservoirs when bitten by phlebotomine sand flies [29]. The southeast of France is endemic for *L. infantum* and more particularly the Alpes-Maritimes (AM), home of the highest incidence in France: 0.64 per 100,000 inhabitants (mean annual incidence in French endemic areas is 0.21 per 100,000 inhabitants) [20]. In the Alpes-Maritimes department, human VL and CanL have been reported with a high proportion of the human population infected asymptomatically [13, 19, 28].

The objective of this study was to investigate asymptomatic leishmaniasis infections in the peripheral blood of healthy humans and their dogs in the AM, using molecular and serological screening techniques.

## Materials and methods

### Ethics statement

Participants were enrolled in the study after written consent was obtained. At the time of the study, written consent was sufficient for the study and there was no regulation for dog sampling.

### “Asymptomatic infection” definition

As highlighted by Ibarra-Meneses *et al*. [18], the definition of asymptomatic *Leishmania* infection is not unified across the literature, but often includes the following criteria: residence (or extended stay) in a *Leishmania* endemic area, no reported signs/symptoms compatible with leishmaniasis, and positive results on a combination of serological, molecular, cellular, and/or parasitological testing methods. In our study, the considered criteria were: residence in a *Leishmania* endemic area (the AM, France), no reported signs/symptoms compatible with leishmaniasis, and positive serological results (Western blot or ELISA) and/or positive molecular tests (kDNA PCR).

### Study site and population studied

**Figure 1:**
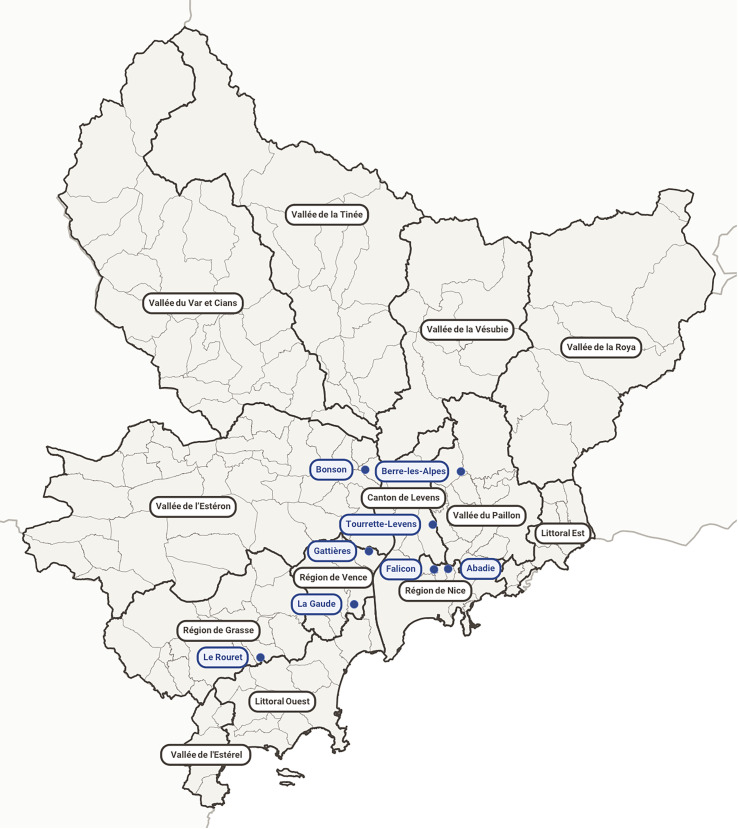
Map with the locations of the campaigns and the population areas used in this study. Population areas usually correspond to a geographic entity (river, valley, plain, urban area, or coastline). In blue, the eight locations where the sampling was performed, and in grey, the population areas (bold lines represent the boundaries of each population area). The place of residence of the participants was assigned to the population areas, as the sampling location does not always correspond to the place of residence.

The study was carried out between 2008 and 2013 in the AM department of southeast France where both human and canine visceral leishmaniases are endemic [26]. Volunteering Campaigns were launched in the following eight locations: Tourette Levens, La Gaude, l’Abadie, Bonson, Le Rouret, Berre les Alpes, Gattières, and Falicon (Fig. 1). Some volunteers resided in different areas than the location of the campaign. In order to estimate the proportion of asymptomatic infections in humans and dogs, the geographic area of the AM was divided into 13 population areas: “Canton de Levens”, “Littoral Est”, “Littoral Ouest”, “Région de Grasse”, “Région de Nice”, “Région de Vence”, “Vallée de l’Estéron”, “Vallée de la Roya”, “Vallée du Paillon”, “Vallée de l’Ésterel”, “Vallée de la Vésubie”, “Vallée de la Tinée”, and “Vallée du Var et Cians”. Data were grouped into population areas that represented the place of residence (Fig. 1). Volunteers (humans and dogs) lived in 12 out of 13 population areas. Only “Vallée de l’Ésterel” was devoid of volunteers.

Sampling periods were from February to April, when the presence of the infected phlebotomine sand flies in the AM was unlikely. The people living in these locations were informed by flyers in their mailbox about the free screening campaign for *Leishmania* serology and PCR. On the day of the campaign, the study was explained for the human participants and their dogs, and written consents were obtained from the dog owners. Peripheral blood sampling of both healthy individuals and their dogs were performed on a voluntary basis. The following data was collected: (i) first name, last name, age, sex, and site of residence of the humans; (ii) name, age, sex, site of residence, length of hair, and breed of the dogs. Participants were given the choice of being sampled alone, with their dog, or having only their dog tested.

#### Sample collection

Venous blood from both humans and dogs was collected into two blood collection tubes of 7 mL (one with clot activator for sera, and one with EDTA for PCR) for serological and molecular analysis. Tubes were stored at 4 °C.

#### Serology

ELISA was performed on canine sera (in house laboratory developed test). Briefly, plates were coated with antigens obtained from a lysate of culture of promastigotes of *L. infantum*. Dog sera were 1/200 diluted then incubated for 30 min at 37 °C, washed with phosphate buffered saline solution and incubated with a secondary antibody conjugated to Protein A/G horseradish Peroxidase for 30 min at 37 °C. The ELISA plates were washed with phosphate buffered saline solution, and TMB ELISA peroxidase substrate (Interchim, Montluçon, France) was added for 15 min at 37 °C. After incubation, 0.1 N H_2_SO_4_ was added to the wells and optical density was estimated by a microplate reader (Multiskan EX Type R5232C, ThermoFisher, Waltham, MA, USA). A positive ELISA corresponds to a titer ≥80 IU. An ELISA titer above 400 IU was considered in favor of active CanL.

For human samples, serology was performed by in house Western blot as already described [27]. Briefly, once the nitrocellulose membrane was sensitized with *L. infantum* (MHOM/FR/81/LPN5) antigens, serum was incubated overnight then washed and revealed. Serology was considered positive when bands 14 kDa and/or 18 kDa were present [27].

#### DNA preparation from buffy coat

The 7 mL EDTA collection tubes were centrifuged at 800× *g* for 10 min with the brake off at room temperature. Buffy coats from human and canine blood samples were taken and aliquoted. 200 μL were preserved at −20 °C and were used for DNA extraction and PCR amplification. DNA was extracted using a DNeasy 96 Blood & Tissue Kit (QIAGEN, Courtaboeuf, France) and eluted in 50 μL AE buffer, according to the manufacturer’s instructions. DNA was stored at −20 °C until nested PCR achievement.

#### PCR analysis

Nested PCR was performed to detect parasite DNA in buffy coats with primers targeting the kinetoplast minicircles [32]. In presence of *L. infantum* DNA, a 680 bp product was amplified. For the first PCR, every 30 μL of reaction mix was composed of 10 pMol of each primer (external primers CSB2XF 5′-(GC)(AG)T(AG)CAGAAA(CT)CCCGTTCA-3′ and CSB1XR 5′-ATTTTTC(GC)G(AT)TT(CT)GCAGAACG)-3′), 2 μL of template DNA, 1 nMol of each dNTP, 3 μL of buffer 10X, and 1.5 U of Taq Polymerase (5 U/μL, Roche Diagnostics, Meylan, France). Amplifications were carried out in a thermal cycler (Mastercycler, Eppendorf); using the following conditions: 94 °C for 2 min, followed by 40 cycles of 94 °C for 30 s, 54 °C for 60 s, and 72 °C for 90 s, and a final extension step of 72 °C for 10 min. For the second PCR, every 30 μL of reaction mix was composed of 10 pMol of each primer (internal primers 13Z 5′-ACTGGGGGTTGGTGTAAAATAG-3′ and LiR 5′-TCGCAGAACGCCCCT-3′), 3 μL of PCR1 product (1:10 dilution), 1 nMol of each dNTP, 3 μL of buffer 10X, and 1.5 U of Taq Polymerase (5 U/μL, Roche Diagnostics). Amplifications were carried out in a thermal cycler using the following conditions: 94 °C for 2 min, followed by 40 cycles of 94 °C for 30 s, 56 °C for 60 s, and 72 °C for 40 s; and a final extension step of 72 °C for 10 min. Both negative control (no template) and positive control (10 pg of *L. infantum* MHOM/FR/78/LEM75) were added to each trial. All of the amplification reactions were done in triplicate and a 680 bp fragment, if present, was visualized by 1.5% agarose gel electrophoresis, with EZ-vision staining (Amresco^®^, Solon, OH, USA).

#### Statistical analysis

Student’s *t*-test was used to compare quantitative variables between two groups, if they had a Gaussian distribution; otherwise, the Wilcoxon–Mann–Whitney test was used. The difference in mean, its 95% confidence interval, the standardized difference (Cohen’s *d*), and the percentage variation between groups were calculated. Univariate analyses were done with Pearson’s *χ*^2^ test (or Fisher’s exact test) for qualitative variables, and an analysis of variance (ANOVA) test (or Kruskal–Wallis rank test) for quantitative variables.

All analyses involved two-sided *p*-values, with statistical significance defined by *p* ≤ 0.05. Statistical analyses were performed with R software (version 4.1.2) and the RStudio suite (version 2022.2.0.443).

## Results

### Description of the studied population

During the campaigns, 969 participants were sampled (343 humans and 626 dogs). In total, 1,171 samples were collected, among which 389 samples from humans and 782 from dogs; some participants took part in several sampling campaigns. In order to select only dogs with asymptomatic infection without suspected CanL, 19 seropositive dogs with high antibody titers above 400 units were excluded. At such titers, these dogs could indeed have concussive symptomatic CanL. The data for 607 dogs were taken into account for the analysis, representing 763 samples. In Tourette-Levens, 225 samples were collected, with most samples coming from dogs (23.3% (*n* = 178)), while in l’Abadie and La Gaude, most samples were collected from humans (17% in both villages (*n* = 66)) (Table 1).

**Table 1:** Distribution of dog and human samples in the eight studied locations. Some humans (*n* = 31) and dogs (*n* = 100) were sampled several times in different locations.

Villages *n* (%)	Tourrette-Levens	La Gaude	L’Abadie	Bonson	Le Rouret	Berre les Alpes	Gattières	Falicon	Total
Date	02/02/08	04/05/08	02/14/09	04/04/09	02/06/10	04/24/10	01/15/11	03/23/13	
Dogs	178 (23.3%)	87 (11.4%)	100 (13.1%)	54 (7.1%)	64 (8.4%)	139 (18.2%)	54 (7.1%)	87 (11.4%)	**763**
Humans	47 (12.1%)	66 (17.0%)	66 (17.0%)	45 (11.6%)	55 (14.1%)	42 (10.8%)	21 (5.4%)	47 (12.1%)	**389**
Total	**225 (19.5%)**	**153 (13.3%)**	**166 (14.4%)**	**99 (8.6%)**	**119 (10.3%)**	**181 (15.7%)**	**75 (6.5%)**	**134 (11.6%)**	**1152**

Humans in the study had an age range from 8 to 92 years, with a median of 52 years (interquartile range: 43–62 years). The gender distribution included 182 women and 161 men. In 91% of cases (*n* = 312), a single specimen was collected, while in 9% of cases (*n* = 31) two or more specimens were collected.

Dogs were between 0 (puppy of a few months) and 17 years old, with a median age of 5 years. The number of female and male dogs was the same: 299. For the dog population, 83.5% (*n* = 507) were sampled only once and 16.5% (*n* = 100) were sampled two or more times. There were 63.3% (*n* = 384) short-haired dogs, 18.1% (*n* = 110) medium-long haired, 12.9% (*n* = 78) long-haired, and 5.8% (*n* = 35) missing data. We also classified the dogs according to the United Kennel Club (UKC) classification registry (Table 4).

### Serological and PCR test results in humans and dogs

Serology was positive in 40.2% of humans (*n* = 138/343) and in 9.9% of dogs (*n* = 60/607) (Table 2). For the dogs, ELISA test results were positive on the low side which correlates to asymptomatic infection (the highest ELISA titer was 330 units). PCR was positive for 46.9% (*n* = 161/343) and 18.3% (*n* = 111/607) of humans and dogs, respectively. Altogether, there was 66.2% (*n* = 227/343) of humans and 25.7% (*n* = 156/607) of dogs with positive serology and/or positive PCR. The humans and the dogs with positive serology (Western blot or ELISA) and/or positive molecular tests (kDNA PCR), and no symptoms at the time of sampling, were considered asymptomatic infected participants. None of the human volunteers developed symptomatic leishmaniasis after a follow-up period of 9–14 years.

**Table 2:** Serological and PCR test results in humans and dogs.

	Humans *n* = 343			Dogs *n* = 607		
	Serology			Serology		
PCR	Negative	Positive	Total	Negative	Positive	Total
Negative	116 (33.8%)	66 (19.2%)	182 (53.1%)	451 (74.3%)	45 (7.4%)	496 (81.7%)
Positive	89 (25.9%)	72 (21.0%)	161 (46.9%)	96 (15.8%)	15 (2.5%)	111 (18.3%)
Total	205 (59.8%)	138 (40.2%)		547 (90.1%)	60 (9.9%)	

Among the humans and the dogs that were sampled several times, if serology and/or PCR were positive once, these were considered asymptomatic infected individuals. In this cohort, 67 participants (7 humans and 60 dogs) and 50 other participants (19 humans and 31 dogs) remained negative and positive, respectively, at every sampling time. The remaining 14 participants who tested negative initially tested positive on the second or the third sampling time, either by PCR (6 individuals), serology (6 individuals), or both (2 individuals) (Table 3). Of note, 2 humans and 2 dogs tested negative on the first sampling trial then tested positive on the second one, but retested negative on the third or fourth sampling trials.

**Table 3:** Individuals tested secondarily positive.

Tests	Humans (*n* = 5)	Dogs (*n* = 9)	Totals (*n* = 14)
Serology	0	6	6
PCR	4	2	6
Serology + PCR	1	1	2

Since serology was carried out by different tests, we compared only the results of PCR tests, which showed that humans are statistically more infected 46.9% (*n* = 161/343) than dogs 18.3% (*n* = 111/607) (*χ*^2^ *p* < 0.0001).

### Characteristics of the dog and human population according to their serology and PCR test results

#### Dog population

In dogs, there was a significant association between the UKC classification and the asymptomatic infection status (*p* = 0.04). Scenthounds (31.4% (*n* = 49/156)), gun dogs (25% (*n* = 39/156)) and herding dogs (22.4% (*n* = 35/156)) were more infected than the other dogs (Table 4, *p* = 0.04). Hair length was found to have an impact on the asymptomatic status (Table 4). Shorthaired dogs were more frequently infected (71.8% (*n* = 112/156)) than long-haired dogs (12.2% (*n* = 19/156)) (*p* = 0.043). No association was found between the dog sex, age (median was 5 years in non-infected and asymptomatic dogs), and the asymptomatic status.

**Table 4:** Status of asymptomatic infection in dogs according to the UKC classification and according to hair length.

General characteristics	Non infected dogs *n* = 451	Asymptomatic infected dogs *n* = 156	Total *n* = 607 (%)	*p*-value
Male/Female, *n* (%) (9 missing data)	210 (46.6%)/234 (51.9%)	89 (51.9%)/65 (41.7%)		0.078
Median age	5 (0–17)	5 (0–17)		0.96
UKC classification				0.04
Companion dog	46 (10.2%)	6 (3.8%)	52 (8.6%)	
Guardian dog	34 (7.5%)	14 (9.0%)	48 (7.9%)	
Gun dog	86 (19.1%)	39 (25.0%)	125 (20.6%)	
Herding dog	93 (20.6%)	35 (22.4%)	128 (21.1%)	
Northern breed	7 (1.6%)	2 (1.3%)	9 (1.5%)	
Scenthound	118 (26.2%)	49 (31.4%)	167 (27.5%)	
Sighthound Pariah	7 (1.6%)	3 (1.9%)	10 (1.6%)	
Terrier	32 (7.1%)	4 (2.6%)	36 (5.9%)	
Missing	28 (6.2%)	4 (2.6%)	32 (5.3%)	
Classification according to hair length				0.043
Short-haired	272 (60.3%)	112 (71.8%)	384 (63.3%)	
Medium-long haired	90 (20.0%)	20 (12.8%)	110 (18.1%)	
Long-haired	59 (13.1%)	19 (12.2%)	78 (12.9%)	
Missing data	30 (6.7%)	5 (3.2%)	35 (5.8%)	

Depending on the place of residence, the asymptomatic infection status varied significantly (*p* < 0.001) (Table 5). Considering the population areas where 30 or more dogs were sampled, the highest percentage of asymptomatic infections was found in the “Canton de Levens” and in the “Région de Vence” with 36.9% and 28.2%, respectively. The lowest percentage of asymptomatic infections was found in the “Région de Nice” with 16.2%.

**Table 5:** Asymptomatic infections of dogs and humans according to the population areas.

Locations	Dog population *p* < 0.001			Human population *p* = 0.226		
	Non infected *n* = 451	Asymptomatic infection *n* = 156	Total	Non infected *n* = 116	Asymptomatic infection *n* = 227	Total *n* = 343 (%)
Canton de Levens	**65 (63.1%)**	**38 (36.9%)**	**103**	10 (34.5%)	19 (65.5%)	29
Littoral Est	6 (75.0%)	2 (25%)	8	0 (0%)	2 (100%)	2
Littoral Ouest	16 (57.1%)	12 (42.9%)	28	6 (37.5%)	10 (62.5%)	16
Région de Grasse	**42 (85.7%)**	**7 (14.3%)**	**49**	**26 (47.3%)**	**29 (52.7%)**	**55**
Région de Nice	**100 (85.5%)**	**19 (16.2%)**	**117**	**30 (30.3%)**	**69 (69.7%)**	**99**
Région de Vence	**51 (71.8%)**	**20 (28.2%)**	**71**	**13 (29.5%)**	**31 (70.5%)**	**44**
Vallée de l’Estéron	**42 (93.3%)**	**3 (6.7%)**	**45**	**8 (21.6%)**	**29 (78.4%)**	**37**
Vallée de la Roya	0 (0%)	2 (100%)	2	0 (0%)	2 (100%)	2
Vallée de la Tinée	2 (100%)	0 (0%)	2	0 (0%)	0 (0%)	0
Vallée de la Vésubie	2 (28.6%)	5 (71.4%)	7	0 (0%)	0 (0%)	0
Vallée du Paillon	**124 (71.3%)**	**48 (27.6%)**	**174**	**23 (39.7%)**	**35 (60.3%)**	**58**
Vallée du Var et Cians	1 (100%)	0 (0%)	1	0 (0%)	0 (0%)	0
Vallée de l’Ésterel	NA	NA	NA	NA	NA	NA
Missing data	0 (0%)	0 (0%)	0	0 (0%)	1 (100%)	1

NA means that, in our population, nobody was resident in the area. In bold, population area where 30 or more individuals were sampled.

#### Human population

In humans, there were more asymptomatic infections in men (54.6% (*n* = 124)) than in women (45.4% (*n* = 103)) (*p* < 0.001). There was no statistical difference according to age, even though asymptomatic humans were older (mean of 53 years) than non-infected humans (mean of 50 years). When taking into account the population areas where 30 or more humans were sampled, the highest percentages of asymptomatic infection were found in “Vallée de l’Estéron” and “Région de Vence” with 78.4% and 70.5%, respectively. The lowest percentage of asymptomatic infection was in “Région de Grasse” with 52.7% (Table 5).

In order to assess the geographic distribution of human asymptomatic infections, the proportion was calculated according to population area. Thus, the highest proportion of human asymptomatic infection (population areas with 30 or more humans) was found in “Région de Vence” (4.5/100,000) and “Vallée du Paillon,” (3.4/100,000) (Table 6).

**Table 6:** Proportion of human asymptomatic infections according to the population areas.

Population area	Number of asymptomatic infections	Values/100,000 people
Canton de Levens	19	2.8
Littoral Est	2	0.4
Littoral Ouest	10	0.7
Région de Grasse	29	2.7
**Région de Nice**	**69**	**0.1**
**Région de Vence**	**31**	**4.5**
Vallée de l’Estéron	29	16.0
Vallée de la Roya	2	14.2
Vallée de la Tinée	NA	NA
Vallée de la Vésubie	NA	NA
**Vallée du Paillon**	**35**	**3.4**
Vallée du Var et Cians	NA	NA
Vallée de l’Ésterel	NA	NA
Missing data	1	

NA means that, in our population, nobody was residing in the area. In bold, population area where 30 or more participants were sampled.

#### Analysis of asymptomatic infections by PCR by household

There were 372 households in our studied population of humans and dogs. In 63 out of 372 households, only humans were tested; whereas in 96 households, only dogs were tested (dog owners were not tested). In the remaining 213 households, both humans and dogs were tested, and we found that 10.3% of households (*n* = 22) had only asymptomatic infected dogs, 38.5% (*n* = 82) had only asymptomatic infected humans, 15% (*n* = 32) had both asymptomatic infected dogs and humans, and 36.2% (*n* = 77) were completely negative. Thus, in total 23.4% (*n* = 22 + 32) and 53.5% (*n* = 82 + 32) of these households had dogs and humans asymptomatically infected, respectively. No association was found between asymptomatic infections in dog owners and dog infections.

## Discussion

In the present study, we assessed asymptomatic *L. infantum* infection in healthy dogs and their owners using both molecular and serological tools. We found that 66.2% of humans and 25.7% of dogs were positive by serology and/or PCR.

PCR test results were positive in 46.9% and 18.3% of humans and dogs, respectively. Recent studies using PCR gave similar results with 45% positive tests in humans and 25% in dogs [5, 9]. The lack of correlation between serological (40.2% and 9.9% positivity in humans and dogs, respectively) and PCR results has already been highlighted in several studies [3, 5, 16]. Some studies pointed out that the presence of antibodies may be due to a self-resolved past infection leading to the presence of antibodies without any circulating DNA. In addition, serological tests have different sensitivities making it difficult to compare results from one test to another [23]. Altogether, in order to detect asymptomatic infection, a combination of tests enables better estimation of the prevalence than the use of one single test [33]. Western blot is a highly sensitive test used in our study for the human population, while ELISA tests were used in dogs. Thus, asymptomatic infection detected in dogs by serology is probably underestimated compared to the human population. However, the different results of asymptomatic infection between humans and dogs cannot be explained by the use of different tests, as only PCR test results were compared and showed that there were significantly more asymptomatic infections in humans than in dogs (46.9% and 18.3%).

Depending on the population area (areas with at least 30 sampled individuals), asymptomatic infections ranged from 52.7% to 78.4% in humans and from 6.7% to 39.6% in dogs. The highest percentage of asymptomatic infections in both humans and dogs was in “Région de Vence” with 70.5% and 28.2%, respectively. When focusing on households with both humans and dogs, we also found more asymptomatic infections in humans (53.5%) than in dogs (23.4%). While in humans, gender (more men than women) has a significant impact on the presence of asymptomatic infections, in dogs, no statistical difference was found according to sex. These data in humans were already highlighted in other studies [17, 20, 28]. Some studies found that age has an influence on asymptomatic status in humans [17, 28, 42, 44]. The absence of differences regarding age in our human population may be explained by the median age of 52 years related to a rather long exposure to the bite of a potentially infected phlebotomine sand fly.

In dogs, factors influencing the asymptomatic carrier status were hair length and the UKC classification of dogs (scenthounds (31.4%), gun dogs (25%) and herding dogs (22.4%)). Depending on the length of hair, the phlebotomine sand fly may face an obstacle to bite [31, 40]. An association between dog seropositivity and hair length has also been demonstrated by Selim *et al*. in Egypt [39]. Thus short-haired dogs are more vulnerable to the bite of phlebotomine sand fly and are thus more susceptible to be infected, contrarily to long-haired ones. Long hair in dogs decreases emissions of CO_2_ and heat radiation from the host’s body, making it less appealing to vectors [24]. Other risk factors such as dog median age (5 years) were not associated with asymptomatic infections in our study, contradicting the data described by Selim *et al*. [39]. In addition, in the different UKC categories of dogs, some of them are more at risk than others. Indeed, scenthounds, gun dogs and herding dogs are more frequently involved in hunting or herding, leading to longer exposure to the vector due to their outdoor activities.

In our study, asymptomatic infection was significantly more important in humans than in dogs. This result was inconsistent with the finding of Ferroglio *et al*. who found more infected dogs (42.22%) than humans (16.81%) in the endemic area of northern Italy, close to our study area [12]. However, in the Ferroglio *et al*. study, dogs and humans were sampled independently without relationship of dogs and dog owners, contrary to our study. Several hypotheses could be formulated to explain the higher percentage of asymptomatic infections in humans versus dogs. One explanation could be the use of two different serological methods: Western blot for human samples and ELISA for canine samples. However, when comparing the data obtained by PCR only, humans remained more infected than dogs. The fact that humans have longer exposure to the vector due to a longer life expectancy could also be another explanation. In addition, once infected by *L. infantum*, humans remain mostly asymptomatic carriers and VL may occur in case of immunosuppression thereafter [1, 17, 28]. In contrast, dogs are more prone to develop CanL, even though some dogs are resistant and will never develop any clinical signs upon infection [8, 41]. Another hypothesis could be the circulation of two types of parasites: one infecting mainly dogs leading to CanL and another type of parasite infecting mainly humans, leading to asymptomatic infection. However, studies genetically comparing *L. infantum* strains from humans and dogs have shown that there is no clear host specificity. Specific genotypes were found in domestic animals, wild animals and in humans; however, some of them were shared by all three hosts [12, 34, 35]. This suggests that several parasite transmission cycles co-exist which could be shaped by the structure of the different sand fly populations [34]. Recently, Prudhomme *et al*. demonstrated the presence of two distinct *Phlebotomus ariasi* sand flies (sylvatic and domestic), with distinct genetic structures affected by altitude and hillside [36]. A particular population of sand flies may have an influence on *Leishmania* spp. transmission [36]. Studying the genetic structure of *L. infantum* strains from sand flies and from asymptomatically infected humans is required to validate these hypotheses. However, strains from asymptomatic infected individuals are difficult to obtain in culture, as there are usually very few blood circulating parasites. In addition, there are very few *L. infantum* strains obtained from sand flies of the “Alpes-Maritimes”. More studies on genetic and virulence factors of such strains are needed in order to fully explore the characteristics of *L. infantum* strains infecting humans [13, 28, 34, 35].

Due to a higher percentage of asymptomatic infections in humans than in dogs, one may ask whether a dog owner asymptomatic for *L. infantum* may constitute a risk factor for dog infection. Most of the research studies were designed to study whether dog infection constitutes a risk to humans, but few studied the opposite. Recently, Teixeira *et al*. found that a higher socioeconomic status of dog owners is a risk factor for CanL, suggesting a behavioral link between dog owners and canine infection [40]. In our endemic area, the role of human asymptomatic infection in the transmission cycle of the parasite should be further studied due to their high prevalence. Indeed, asymptomatic infection in humans and dogs could have an impact on parasite transmission if these asymptomatic carriers are found to be infective to phlebotomine sand fly. This has been demonstrated for asymptomatic dogs that are competent to transmit the parasite to phlebotomine sand fly [22, 29, 37, 41]. Thus, they would play a role as reservoir and a role in the transmission cycle of *L. infantum*, which makes the presence of asymptomatic dogs a topic of consideration in control programs of CanL. New, dog friendly, easy-to-perform diagnostic tests, with good sensitivity and specificity, should be used in order to detect infected dogs as soon as possible. Once tested positive, these dogs should be protected from the phlebotomine sand fly bite. Two main methods are commonly used: dog collars and spot-on insecticides. Some dog owners also use insecticide spraying of the kennel. To date, dog vaccination against *L. infantum* does not protect from the infection and it is recommended to use topical insecticide simultaneously [43]. In humans, studies are scarce on the infectious potential of asymptomatic carriers to phlebotomine sand fly. Feirrera *et al*. conducted xenodiagnoses on non-HIV human asymptomatic carriers [11]. None of the phlebotomine sand flies were found to be infected using microscopy, but some (27 out of the 760) were found to be positive by PCR. In Molina *et al*., the authors found that human asymptomatic carriers do not play an important role in the transmission of *L. infantum* in the Mediterranean region, using indirect xenodiagnosis [30]. As stated by the authors, there is no clear evidence that human asymptomatic carriers are infectious to phlebotomine sand fly, so the question whether asymptomatic carriers are infective to phlebotomine sand fly remains open.

## References

[1] Akuffo H, Costa C, van Griensven J, Burza S, Moreno J, Herrero M. 2018. New insights into leishmaniasis in the immunosuppressed. PLoS Neglected Tropical Diseases, 12, e0006375. 29746470 10.1371/journal.pntd.0006375 PMC5944929

[2] Alborzi A, Pourabbas B, Shahian F, Mardaneh J, Pouladfar GR, Ziyaeyan M. 2008. Detection of *Leishmania infantum* kinetoplast DNA in the whole blood of asymptomatic individuals by PCR-ELISA and comparison with other infection markers in endemic areas, southern Iran. American Journal of Tropical Medicine and Hygiene, 79, 839–842. 19052289

[3] Aliaga L, Ceballos J, Sampedro A, Cobo F, López-Nevot MÁ, Merino-Espinosa G, Morillas-Márquez F, Martín-Sánchez J. 2019. Asymptomatic *Leishmania* infection in blood donors from the Southern of Spain. Infection, 47, 739–747. 30888587 10.1007/s15010-019-01297-3

[4] Asfaram S, Fakhar M, Mohebali M, Mardani A, Banimostafavi ES, Ziaei Hezarjaribi H, Soosaraei M. 2017. Asymptomatic human blood donors carriers of *Leishmania infantum*: Potential reservoirs for visceral leishmaniasis in northwestern Iran. Transfusion and Apheresis Science, 56, 474–479. 28648574 10.1016/j.transci.2017.06.001

[5] Asfaram S, Fakhar M, Mohebali M, Ziaei Hezarjaribi H, Mardani A, Ghezelbash B, Akhoundi B, Zarei Z, Moazeni M. 2022. A Convenient and sensitive kDNA-PCR for screening of *Leishmania infantum* latent infection among blood donors in a highly endemic focus, Northwestern Iran. Acta Parasitologica, 67, 842–850. 35294973 10.1007/s11686-022-00528-2

[6] Bañuls AL, Bastien P, Pomares C, Arevalo J, Fisa R, Hide M. 2011. Clinical pleiomorphism in human leishmaniases, with special mention of asymptomatic infection. Clinical Microbiology and Infection, 17, 1451–1461. 21933304 10.1111/j.1469-0691.2011.03640.x

[7] Costa CHN, Stewart JM, Gomes RBB, Garcez LM, Ramos PKS, Bozza M, Satoskar A, Dissanayake S, Santos RS, Silva MRB, Shaw JJ, David JR, Maguire JH. 2002. Asymptomatic human carriers of *Leishmania chagasi*. American Journal of Tropical Medicine and Hygiene, 66, 334–337. 12164285 10.4269/ajtmh.2002.66.334

[8] Esteva L, Vargas C, Vargas de León C. 2017. The role of asymptomatics and dogs on leishmaniasis propagation. Mathematical Biosciences, 293, 46–55. 28864398 10.1016/j.mbs.2017.08.006

[9] Fakhar M, Derakhshani-Nia M, Gohardehi S, Karamian M, Hezarjaribi HZ, Mohebali M, Akhoundi B, Sharbatkhori M. 2022. Domestic dogs carriers of *Leishmania infantum*, *Leishmania tropica* and *Crithidia fasciculata* as potential reservoirs for human visceral leishmaniasis in northeastern Iran. Veterinary Medicine and Science, 8, 2329–2336. 36063538 10.1002/vms3.929 PMC9677403

[10] Faucher B, Pomares C, Fourcade S, Benyamine A, Marty P, Pratlong L, Faraut F, Mary C, Piarroux R, Dedet J-P, Pratlong F. 2011. Mucosal *Leishmania infantum* leishmaniasis: specific pattern in a multicentre survey and historical cases. Journal of Infection, 63, 76–82. 21658772 10.1016/j.jinf.2011.03.012

[11] Ferreira GR, Castelo Branco Ribeiro JC, Meneses Filho A, de Jesus Cardoso Farias Pereira T, Parente DM, Pereira HF, Carlos da Silva J, Zacarias DA, Vieira da Silva L, Medeiros Faustino SK, Almeida Neto WS, Costa DL, Lopes de Mendonça I, Nery Costa CH. 2018. Human competence to transmit *Leishmania infantum* to *Lutzomyia longipalpis* and the Influence of human immunodeficiency virus infection. American Journal of Tropical Medicine and Hygiene, 98, 126–133. 29141704 10.4269/ajtmh.16-0883 PMC5928688

[12] Ferroglio E, Battisti E, Zanet S, Bolla C, Concialdi E, Trisciuoglio A, Khalili S, Biglino A. 2018. Epidemiological evaluation of *Leishmania infantum* zoonotic transmission risk in the recently established endemic area of Northwestern Italy. Zoonoses and Public Health, 65, 675–682. 29745468 10.1111/zph.12477

[13] le Fichoux Y, Quaranta JF, Aufeuvre JP, Lelievre A, Marty P, Suffia I, Rousseau D, Kubar J. 1999. Occurrence of *Leishmania infantum* parasitemia in asymptomatic blood donors living in an area of endemicity in southern France. Journal of Clinical Microbiology, 37, 1953–1957. 10325353 10.1128/jcm.37.6.1953-1957.1999 PMC84994

[14] García-Castro A, Egui A, Thomas MC, López MC. 2022. Humoral and cellular immune response in asymptomatic dogs with visceral leishmaniasis: A review. Vaccines, 10, 947. 35746555 10.3390/vaccines10060947 PMC9229064

[15] Giraldo-Martínez LA, Petano-Duque JM, Uribe-García HF, Chacón-Novoa RA, Guzmán-Barragán BL, Rondón-Barragán I. 2022. High prevalence of *Leishmania* spp. in dogs from Central West Colombia. Veterinaria Italiana, 58, 379–389. 10.12834/VetIt.2632.16820.2 37303138

[16] Guillén MC, Alcover MM, Borruel N, Sulleiro E, Salvador F, Berenguer D, Herrera-de Guise C, Rodríguez V, Moure Z, Sánchez-Montalvà A, Molina I, Fisa R, Riera C. 2020. *Leishmania infantum* asymptomatic infection in inflammatory bowel disease patients under anti-TNF therapy. Heliyon, 6, e03940. 32420499 10.1016/j.heliyon.2020.e03940 PMC7218013

[17] Ibarra-Meneses AV, Carrillo E, Nieto J, Sánchez C, Ortega S, Estirado A, Latasa Zamalloa P, Sanz JC, García-Comas L, Ordobás M, Moreno J. 2019. Prevalence of asymptomatic *Leishmania* infection and associated risk factors, after an outbreak in the south-western Madrid region, Spain, 2015. Eurosurveillance, 24, 1800379. 31164191 10.2807/1560-7917.ES.2019.24.22.1800379 PMC6549460

[18] Ibarra-Meneses AV, Corbeil A, Wagner V, Onwuchekwa C, Fernandez-Prada C. 2022. Identification of asymptomatic *Leishmania* infections: A scoping review. Parasites & Vectors, 15, 5. 34983616 10.1186/s13071-021-05129-y PMC8727076

[19] Lachaud L, Chabbert E, Dubessay P, Dereure J, Lamothe J, Dedet JP, Bastien P. 2002. Value of two PCR methods for the diagnosis of canine visceral leishmaniasis and the detection of asymptomatic carriers. Parasitology, 125, 197–207. 12358417 10.1017/s0031182002002081

[20] Lachaud L, Dedet JP, Marty P, Faraut F, Buffet P, Gangneux JP, Ravel C, Bastien P, Working Group for the Notification of Human Leishmanioses in France. 2013. Surveillance of leishmaniases in France, 1999 to 2012. Euro Surveillance, 18, 20534. 23929121

[21] Lachaud L, Marchergui-Hammami S, Chabbert E, Dereure J, Dedet JP, Bastien P. 2002. Comparison of six PCR methods using peripheral blood for detection of canine visceral leishmaniasis. Journal of Clinical Microbiology, 40, 210–215. 11773118 10.1128/JCM.40.1.210-215.2002 PMC120090

[22] Laurenti MD, Rossi CN, da Matta VLR, Tomokane TY, Corbett CEP, Secundino NFC, Pimenta PFP, Marcondes M. 2013. Asymptomatic dogs are highly competent to transmit *Leishmania* (*Leishmania*) *infantum chagasi* to the natural vector. Veterinary Parasitology, 196, 296–300. 23562649 10.1016/j.vetpar.2013.03.017

[23] Lévêque MF, Battery E, Delaunay P, Lmimouni BE, Aoun K, L’Ollivier C, Bastien P, Mary C, Pomares C, Fillaux J, Lachaud L. 2020. Evaluation of six commercial kits for the serological diagnosis of Mediterranean visceral leishmaniasis. PLoS Neglected Tropical Diseases, 14, e0008139. 32210438 10.1371/journal.pntd.0008139 PMC7135331

[24] Lopes PM, Sorte EDCB, Gasparetto ND, Oliveira CM, Almeida ADBPFD, Sousa VRF. 2014. Seroprevalence and risk factors associated with visceral leishmaniasis in dogs in Jaciara, State of Mato Grosso. Revista da Sociedade Brasileira de Medicina Tropical, 47, 791–795. 25626662 10.1590/0037-8682-0027-2014

[25] Martín-Sánchez J, Pineda JA, Morillas-Márquez F, García-García JA, Acedo C, Macías J. 2004. Detection of *Leishmania infantum* kinetoplast DNA in peripheral blood from asymptomatic individuals at risk for parenterally transmitted infections: relationship between polymerase chain reaction results and other Leishmania infection markers. American Journal of Tropical Medicine and Hygiene, 70, 545–548. 15155989

[26] Marty P, Izri A, Ozon C, Haas P, Rosenthal E, Del Giudice P, Godenir J, Coulibaly E, Gari-Toussaint M, Delaunay P, Ferrua B, Haas H, Pratlong F, Le Fichoux Y. 2007. A century of leishmaniasis in Alpes-Maritimes, France. Annals of Tropical Medicine and Parasitology, 101, 563–574. 17877875 10.1179/136485907X229121

[27] Marty P, Lelievre A, Quaranta JF, Rahal A, Gari-Toussaint M, Le Fichoux Y. 1994. Use of the leishmanin skin test and western blot analysis for epidemiological studies in visceral leishmaniasis areas: experience in a highly endemic focus in Alpes-Maritimes (France). Transactions of the Royal Society of Tropical Medicine and Hygiene, 88, 658–659. 7886761 10.1016/0035-9203(94)90214-3

[28] Michel G, Pomares C, Ferrua B, Marty P. 2011. Importance of worldwide asymptomatic carriers of *Leishmania infantum* (*L. chagasi*) in human. Acta Tropica, 119, 69–75. 21679680 10.1016/j.actatropica.2011.05.012

[29] Molina R, Amela C, Nieto J, San-Andrés M, González F, Castillo JA, Lucientes J, Alvar J. 1994. Infectivity of dogs naturally infected with *Leishmania infantum* to colonized *Phlebotomus perniciosus*. Transactions of the Royal Society of Tropical Medicine and Hygiene, 88, 491–493. 7570854 10.1016/0035-9203(94)90446-4

[30] Molina R, Jiménez M, García-Martínez J, San Martín JV, Carrillo E, Sánchez C, Moreno J, Alves F, Alvar J. 2020. Role of asymptomatic and symptomatic humans as reservoirs of visceral leishmaniasis in a Mediterranean context. PLoS Neglected Tropical Diseases, 14, e0008253. 32324738 10.1371/journal.pntd.0008253 PMC7200008

[31] Monteiro FM, Machado AS, Rocha-Silva F, Assunção CB, Graciele-Melo C, Costa LE, Portela AS, Ferraz Coelho EA, Maria de Figueiredo S, Caligiorne RB. 2019. Canine visceral leishmaniasis: Detection of *Leishmania* spp. genome in peripheral blood of seropositive dogs by real-time polymerase chain reaction (rt-PCR). Microbial Pathogenesis, 126, 263–268. 30419342 10.1016/j.micpath.2018.10.036

[32] Noyes HA, Reyburn H, Bailey JW, Smith D. 1998. A nested-PCR-based schizodeme method for identifying *Leishmania* kinetoplast minicircle classes directly from clinical samples and its application to the study of the epidemiology of *Leishmania tropica* in Pakistan. Journal of Clinical Microbiology, 36, 2877–2881. 9738037 10.1128/jcm.36.10.2877-2881.1998 PMC105081

[33] Ortalli M, De Pascali AM, Longo S, Pascarelli N, Porcellini A, Ruggeri D, Randi V, Procopio A, Re MC, Varani S. 2020. Asymptomatic *Leishmania infantum* infection in blood donors living in an endemic area, northeastern Italy. Journal of Infection, 80, 116–120. 31585188 10.1016/j.jinf.2019.09.019

[34] Ortuño M, Latrofa MS, Iborra MA, Pérez-Cutillas P, Bernal LJ, Risueño J, Muñoz C, Bernal A, Sánchez-Lopez PF, Segovia M, Annoscia G, Maia C, Cortes S, Campino L, Otranto D, Berriatua E. 2019. Genetic diversity and phylogenetic relationships between *Leishmania infantum* from dogs, humans and wildlife in south-east Spain. Zoonoses and Public Health, 66, 961–973. 31512370 10.1111/zph.12646

[35] Pomares C, Marty P, Bañuls AL, Lemichez E, Pratlong F, Faucher B, Jeddi F, Moore S, Michel G, Aluru S, Piarroux R, Hide M. 2016. Genetic diversity and population structure of *Leishmania infantum* from Southeastern France: Evaluation using multi-locus microsatellite typing. PLoS Neglected Tropical Diseases, 10, e0004303. 26808522 10.1371/journal.pntd.0004303 PMC4726517

[36] Prudhomme J, De Meeûs T, Toty C, Cassan C, Rahola N, Vergnes B, Charrel R, Alten B, Sereno D, Bañuls A-L. 2020. Altitude and hillside orientation shapes the population structure of the *Leishmania infantum* vector *Phlebotomus ariasi*. Scientific Reports, 10, 14443. 32879357 10.1038/s41598-020-71319-w PMC7468129

[37] Quinnell RJ, Courtenay O. 2009. Transmission, reservoir hosts and control of zoonotic visceral leishmaniasis. Parasitology, 136, 1915–1934. 19835643 10.1017/S0031182009991156

[38] Riera C, Fisa R, Udina M, Gállego M, Portus M. 2004. Detection of *Leishmania infantum* cryptic infection in asymptomatic blood donors living in an endemic area (Eivissa, Balearic Islands, Spain) by different diagnostic methods. Transactions of the Royal Society of Tropical Medicine and Hygiene, 98, 102–110. 14964810 10.1016/s0035-9203(03)00015-4

[39] Selim A, Shoulah S, Abdelhady A, Alouffi A, Alraey Y, Al-Salem WS. 2021. Seroprevalence and risk factors associated with canine leishmaniasis in Egypt. Veterinary Sciences, 8, 236. 34679066 10.3390/vetsci8100236 PMC8541007

[40] Teixeira AIP, Silva DM, de Freitas LRS, Romero GAS. 2020. A cross-sectional approach including dog owner characteristics as predictors of visceral leishmaniasis infection in dogs. Memórias do Instituto Oswaldo Cruz, 115, e190349. 32348406 10.1590/0074-02760190349 PMC7184770

[41] de Vasconcelos TCB, Furtado MC, Belo VS, Morgado FN, Figueiredo FB. 2019. Canine susceptibility to visceral leishmaniasis: A systematic review upon genetic aspects, considering breed factors and immunological concepts. Infection, Genetics and Evolution, 74, 103293. 10.1016/j.meegid.2017.10.005 28987807

[42] Velez R, Ballart C, Domenech E, Abras A, Fernández-Arévalo A, Gómez SA, Tebar S, Muñoz C, Cairó J, Gállego M. 2019. Seroprevalence of canine *Leishmania infantum* infection in the Mediterranean region and identification of risk factors: The example of North-Eastern and Pyrenean areas of Spain. Preventive Veterinary Medicine, 162, 67–75. 30621900 10.1016/j.prevetmed.2018.10.015

[43] Velez R, Gállego M. 2020. Commercially approved vaccines for canine leishmaniosis: a review of available data on their safety and efficacy. Tropical Medicine & International Health, 25, 540–557. 32034985 10.1111/tmi.13382

[44] Yimam Ayene Y, Mohebali M, Hajjaran H, Akhoundi B, Shojaee S, Rahimi-Foroushani A, Afshar MJA, Zarei Z. 2021. A comparative study of nested-PCR and direct agglutination test (DAT) for the detection of *Leishmania infantum* infection in symptomatic and asymptomatic domestic dogs. BMC Research Notes, 14, 270. 34256817 10.1186/s13104-021-05654-0 PMC8276487

